# FAIR African brain data: challenges and opportunities

**DOI:** 10.3389/fninf.2025.1530445

**Published:** 2025-03-03

**Authors:** Eberechi Wogu, George Ogoh, Patrick Filima, Barisua Nsaanee, Bradley Caron, Franco Pestilli, Damian Eke

**Affiliations:** ^1^Department of Anatomy, University of Port Harcourt, Port Harcourt, Nigeria; ^2^School of Computer Science, University of Nottingham, Nottingham, United Kingdom; ^3^Department of Psychology and Neuroscience, The University of Texas at Austin, Austin, TX, United States

**Keywords:** FAIR, African neuroscience, brain data, neuroimaging, neuroinformatics

## Abstract

**Introduction:**

The effectiveness of research and innovation often relies on the diversity or heterogeneity of datasets that are Findable, Accessible, Interoperable and Reusable (FAIR). However, the global landscape of brain data is yet to achieve desired levels of diversity that can facilitate generalisable outputs. Brain datasets from low-and middle-income countries of Africa are still missing in the global open science ecosystem. This can mean that decades of brain research and innovation may not be generalisable to populations in Africa.

**Methods:**

This research combined experiential learning or experiential research with a survey questionnaire. The experiential research involved deriving insights from direct, hands-on experiences of collecting African Brain data in view of making it FAIR. This was a critical process of action, reflection, and learning from doing data collection. A questionnaire was then used to validate the findings from the experiential research and provide wider contexts for these findings.

**Results:**

The experiential research revealed major challenges to FAIR African brain data that can be categorised as socio-cultural, economic, technical, ethical and legal challenges. It also highlighted opportunities for growth that include capacity development, development of technical infrastructure, funding as well as policy and regulatory changes. The questionnaire then showed that the wider African neuroscience community believes that these challenges can be ranked in order of priority as follows: Technical, economic, socio-cultural and ethical and legal challenges.

**Conclusion:**

We conclude that African researchers need to work together as a community to address these challenges in a way to maximise efforts and to build a thriving FAIR brain data ecosystem that is socially acceptable, ethically responsible, technically robust and legally compliant.

## 1 Introduction

In the last two decades, advanced machine learning, deep learning, artificial intelligence and the increasing availability of big data have converged to revolutionise global brain research and innovation. From basic and clinical brain research to translational neurotechnology, this convergence has informed epochal advancements in the way data is collected, processed and applied. The age of “big brain data research” where data-intensive/data-driven approaches are opening up new ways of understanding brain development, functions, structures and diseases is here according to Landhuis (2017). This ongoing data-driven cultural transition is characterised by the increasing generation and application of multimodal, multidimensional, multi-functional and cross-border datasets from multiple organisms (human and non-human) and also includes an array of technical data that are made Findable, Accessible, Interoperable and Reusable (FAIR). Facilitating FAIR globally are open data repositories that are growing in number and importance.

The availability of FAIR data is a critical factor that drives research and innovation (Wilkinson et al., 2016). The effectiveness of research and innovation often relies on the diversity or heterogeneity of datasets that are FAIR. However, the global landscape of brain data is yet to achieve desired levels of diversity. Brain datasets from low-and middle-income countries of Africa are still missing in the global research ecosystem (Wogu et al., 2023). Global brain research outputs and neurotechnologies are largely informed by datasets collected from populations in the global north. The scientific and translational implication of this is that the therapies, innovations and other outcomes produced may not be generalisable to populations in Africa. The critical question therefore is: *why are African brain datasets not yet FAIR for global research?*

As Abd-Allah et al. (2016) mentioned, “there are limited data on the contribution of the African continent to neuroscience research and publications.” After reviewing two decades of neuroscience research in Africa, Maina et al. (2021) confirmed this and declared that “neuroscience research in Africa remains sparse.” Whilst the current state of neuroscience research in Africa is experiencing more collaborative networks and international partnerships which are crucial in advancing education and research infrastructure, challenges persist in the African neuroscience landscape (Aderinto et al., 2023). This paper explores the technical, social-cultural, ethical and legal challenges that form barriers to making African brain data FAIR. Through an empirical research approach, we present insights into diverse challenges and opportunities of FAIR African brain data for the advancement of research and innovation in Africa as well as the world in general. The analysis concludes that multiple factors contribute to the lack of availability of African brain data including socio-cultural, legal, technical and scientific factors. It then proposes pathways to effective inclusion of datasets, skills and resources from Africa into the global brain research and innovation activities.

This paper makes contributions to literature by providing empirically sound insights that enriches FAIR data discourse in the global south. These insights are likely to be of interest to researchers that may want to generate, process, apply and share brain data in and from Africa. By identifying both the challenges and possible ways of mitigating the challenges, we contribute directly to the advancement of FAIR brain data in Africa. This micro advancement of FAIR in Africa subsequently contributes to the macro global discourse on FAIR brain data. This is particularly important given that pre-pandemic figures show that across the African Region, more than 116 million people were already estimated to be living with mental health conditions (WHO, 2022). Also, according to Greene et al. (2021) the lifetime prevalence of psychiatric disorders in Africa ranges from 3.3 to 9.8% for mood disorders, from 5.7 to 15.8% for anxiety disorders, from 3.7 to 13.3% for substance use disorders, and from 1.0 to 4.4% for psychotic disorders. Advancing FAIR brain data in Africa will facilitate the development of region-specific approaches to mitigating the burden of these diseases considering genetic, environmental and cultural factors unique to Africa.

## 2 Brain data in Africa: the state of the art

Research in neuroscience remains largely dominated by countries in the global north with the growing number of big brain research projects (Quaglio et al., 2021). Europe, the United States, Canada, Australia, China, Japan and South Korea are among regions and countries who have invested significantly large funds on large-scale brain projects (Adams et al., 2020). With the help of increasing advancements of technology and its significant integration into neuroscience research, these projects are focused on tackling complex neural questions with profound implications for society. In the global south, particularly in Africa, the picture looks different. There are no large-scale brain projects ongoing in Africa. However, there is a growing ecosystem of brain data in Africa. This is demonstrable from about 5,219 Africa’s neuroscience publications since 1996 as presented in recent research by Maina et al. (2021). This study showed that five countries (Nigeria, Egypt, South Africa, Morocco and Tunisia) account for 75% of these publications while there are notable contributions from Kenya, Ethiopia, Tanzania, Cameroon, Algeria, Senegal, Uganda and Ghana. Despite this increasing level of publication of research outputs, the datasets generated and used for the research remain largely siloed and unshared. The implication is that African datasets are currently missing from the available pool of datasets that inform global neuroscience efforts. This is a sad reality given that Africa has the greatest genetic diversity which is critical in the understanding of human health and diseases (Gurdasani et al., 2015; Jorde et al., 2000).

Our definition of data in this paper transcends the historical understanding of data as raw measurements of nervous system structure, operational properties and function to include derived data as well as metadata that describe the full set of processing steps and analyses used to produce the data (Eke et al., 2021). This includes raw data collected from organisms (including human and non-human animals) as well as computational models generated from them. Raw brain data from organisms are generated via a variety of techniques including genetic, molecular, and cellular approaches as well as imaging, physiological, and electrophysiological approaches, laboratory analysis, audio and visual recordings, and behavioural observations. Models on the other hand are computational representations of the brain structures, functions, and networks. As at the time of writing this paper, an examination of available neuroscience research databases or repositories such as ebrains.eu, openneuro, show that brain datasets collected from Africa cannot be found. Whereas some institutional repositories exist (such as the University of Cape Town’s research repository)^1^ to promote Open data efforts, brain datasets remain non-findable, inaccessible, not interoperable and non-reusable (unFAIR). This apparent dearth of African datasets available for global neuroscience research and innovation inform the motivation to write this paper highlighting key challenges and opportunities.

## 3 Methodology

This paper draws from the experiences of the authors in searching for African datasets for research and their positive efforts to collect and share African brain data. This is called *experiential research.* Relying heavily on David Kolb’s theory of learning (Kolb, 1984), experiential research emphasises learning by doing. Experiential methodology is often used in research and teaching and is based on the principle that individuals learn and generate knowledge best via direct experience, reflection, and application (Grant et al., 2001). It involves either the researchers or the participants engaging in meaningful activities or real-world scenarios, followed by critical reflection to deepen understanding and facilitate personal or collective transformation. This approach emphasises active participation and connects theoretical concepts to practical contexts (Batat, 2023).

For several years, we (the authors) failed to identify African brain datasets from available databases/repositories for research, education and training purposes. To address this, we set out on an effort to identify and collect data from multiple imaging centres. This meant that we as researchers became active participants in the data collection process to understand why African brain datasets were not available for global research. The overall aim was to collect, process and share this data via *brainlife.io* (RRID: SCR_020940) as a proof of what is possible with African brain data. The observations of the researchers during this process were then collated and analysed. This process gave us great insights into the challenges and opportunities FAIR African brain data faces. In our perspective, these challenges could be grouped as: technical, socio-cultural, economic and ethical and legal challenges. These categories were identified via the experiential research process.

To validate these insights, observations or reflections, we set out on primary research involving collection of views of researchers across Africa along these categories of challenges. Understanding the challenges and opportunities of FAIR African brain data requires views from wider and key stakeholders from different countries and regions of Africa as well as from diverse disciplines within the neuroscience ecosystem. Thus, a virtual or online questionnaire was utilised to validate our experiences and gain more relevant perspectives. The choice of an online survey or questionnaire is informed by the need to achieve wide geographical reach because Africa is a vast continent with stakeholders in neuroscience spread across different countries. Observations made during experiential activities can be subjective or context-specific since this experiential research took place in one country-Nigeria. A questionnaire allowed us to gather additional data, to measure if the observations align with participants’ reported experiences or perceptions. In essence, whilst experiential research is qualitative, quantitative data from questionnaires can validate trends observed in experiential settings and strengthen the overall analysis.

Google form was used to create the survey, and dissemination was done via existing Africa-wide research networks [including African Brain Data Network (ABDN), Society Of Neuroscientists of Africa (SONA)] as well as on social media platforms (LinkedIn and X). A total of 59 responses were received - 15 females and 44 males. The respondents described their disciplines as neuroscience, neuroanatomy, biomedical sciences, computer sciences, humanities and psychiatry. A total of 25 identified themselves as students, 20 as early career researchers, 10 midcareer and 4 senior career researchers. Datasets respondents are currently working on include behavioural data, neuroimaging (MRI, fMRI, EEG) and genomic data (see Table 1). Responses included both qualitative and quantitative data and these were analysed using descriptive statistical analysis. Responses were received from 18 countries from the five regions of Africa (see Table 1). This research was approved by the Research Ethics Committee of the University Of Port-Harcourt, Nigeria with the reference code: UPH/CERMAD/REC/MM84/056.

**Table 1:** Demographic distribution of respondents.

Gender	15 females and 44 males
Disciplines	Neuroscience, Neuroanatomy, Biomedical sciences, Computer sciences, Humanities and Psychiatry.
Career stages	25 Students, 20 Early career researchers, 10 Mid-career researchers, 4 senior career researchers
Types of data currently used	Behavioural data, Neuroimaging (MRI, fMRI, EEG) and Genomic data.
Countries	18 African Countries (Nigeria, Rwanda, Somalia, South Africa, Sudan, Togo, Tunisia, Uganda, Zambia, Zimbabwe, Algeria, Botswana, Democratic Republic of Congo, Egypt, Ethiopia, Gambia, Ghana and Mali)
Regions	5 regions in Africa (Northern Africa, West Africa, East Africa, Central Africa and Southern Africa).

## 4 Findings

### 4.1 Challenges to FAIR brain data in Africa

**Figure 1:**
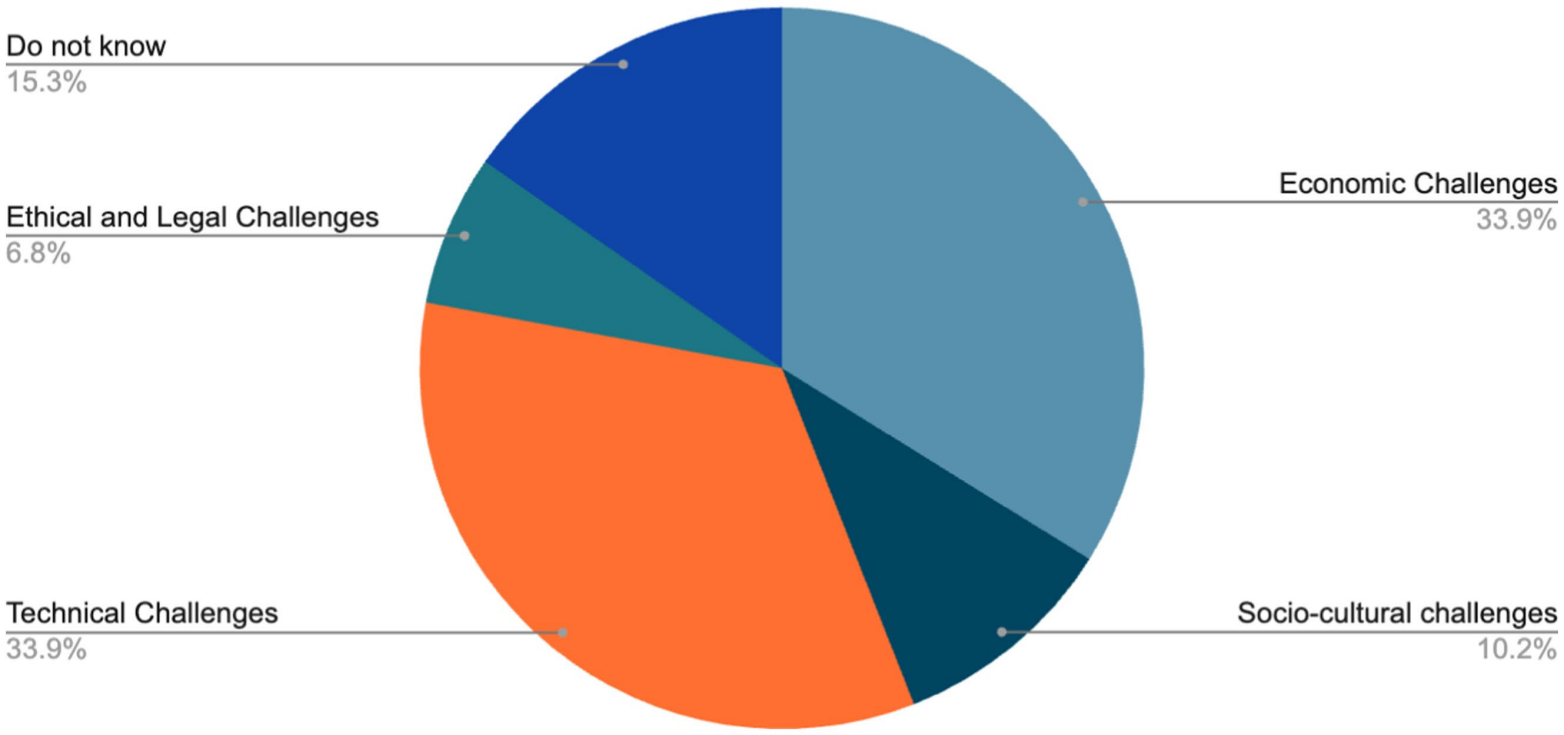
Challenges to FAIR African brain data.

The challenges identified in the process of making this pilot use case FAIR are categorised into sociocultural, economic, technical, ethical, legal and psychological challenges. This is because brain data generation, processing and sharing required for FAIR demands a number of things, necessary knowledge, skills, resources and tools, adequate and sufficient infrastructure and conducive socio-cultural and legal environment. As mentioned above, these challenges are drawn from the experiences of the researchers in obtaining the data, processing, curation and data sharing. According to the findings, the most critical challenges to FAIR brain data in Africa are technical (33.9%) and economic challenges (33.9%). Interestingly 15.3% did not know while 10.2% voted socio-cultural challenges as the third most critical. Ethical and legal challenges are the least critical challenge according to the findings (6.8%). The views of the respondents on these challenges are thus presented below in Figure 1.

#### 4.1.1 Technical challenges

Many of the participants observed that their major challenges are *“inadequacy of modern neuro-behavioural evaluation equipment,” “lack of facilities and expertise” and “facilities and expertise for large dataset acquisition are not available.”* These direct quotes corroborate the insights we got from our data collection exercise. One participant captured it this way; *“I believe the manual approach to gathering, collecting, storing the available data as a field as against digitised methods coupled with not having central (national, regional and continental) repositories like other developed nations has not helped matters.”* These views validate what we found as we were collecting data. In our neuroimaging data collection exercise, one striking challenge was the scarcity of MRI scanning machines. At the time of completing this research, it should be noted that there is only one dedicated research-dedicated Magnetic Resonance (MRI) scanner in Africa - situated at the University of Cape Town South Africa. All available machines are for clinical purposes and even these are difficult to access for research. This lack of research scanners is a major problem because owing to both ethical and legal issues clinical datasets are more difficult to be made FAIR.

In Africa, even the clinical scanners are difficult to come by. For instance, within the six geopolitical zones in Nigeria where the neuroimaging data collection took place, the number of functioning MRI scanners per zone ranges from 1 to 4 and these scanners are only situated at the metropolitan cities in each zone with the South-western geopolitical zone having the highest number of MRI scanners while some other zones have about 1–3 MRI scanners. In the South–South zone of Nigeria which consists of a minimum of 6 states, only about three MRI scanners were found in good working condition with some states not having a single MRI scanner. Hence patients often embark on interstate travels to have access to an MRI facility. This delays access, creates inconvenience and possibly discourages patients from seeking such services.

**Figure 2:**
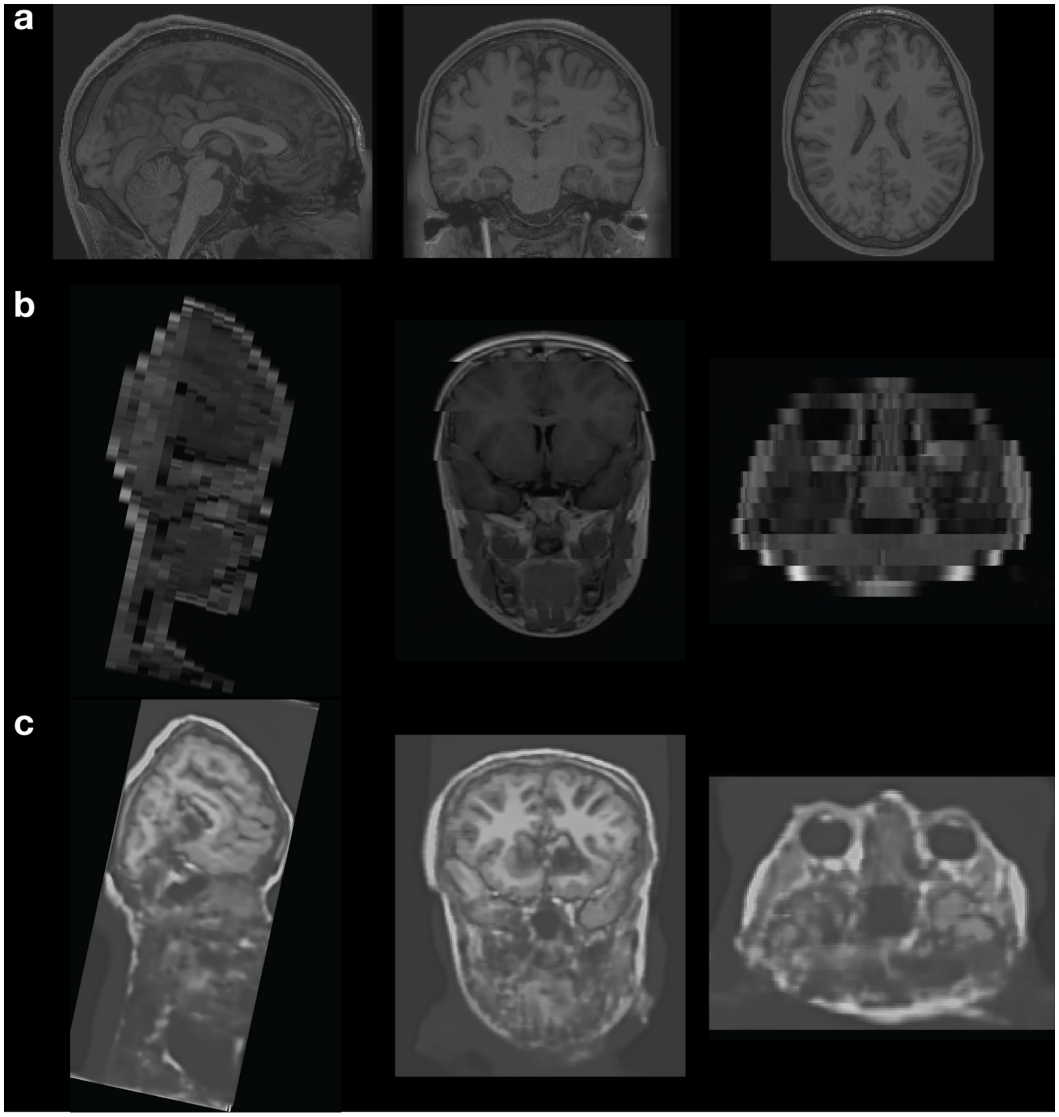
Comparison of research-quality (HCP) and clinical-quality Nigerian brain data and failure of modern AI tools. Demonstration of the difference in quality (spatial resolution and contrast) between research-quality anatomical (T1w) data from the Human Connectome Project (HCP) and data collected from the Nigerian Data Project (Wogu et al., 2023). Attempts to use AI tools to synthesize research-quality 3D data from the clinical data failed to accurately correct for issues in the clinical data to synthesize research-quality data. **(A)** Anatomical (T1w) image from the Human Connectome Project (HCP). **(B)** Anatomical (T1w) image collected from the Nigerian Data Project (Wogu et al., 2023). **(C)** Synthesized 3D anatomical (T1w) image using *SynthSR* [“an AI tool that takes clinical brain MRI scans with any MR contrast (T1, T2, etc.), orientation (axial/coronal/sagittal), and resolution and turns them into high-resolution T1 scans that are usable by virtually all existing human neuroimaging tools”] (Iglesias et al., 2023).

Diagnostic centres are therefore often the primary source of MRI data in Africa. The scanners are of older technology and often do not meet the modern standards for research MRI. For instance, of the three collection sites we used in Nigeria during the experiential approach, two have 1.5 Tesla (T) MRI units and one has 0.3 Tesla (T) units. In comparison, the standard MRI scanner for research in the high-income countries is 3 T or 7 T which can provide better quality image, higher spatial resolution and more complex MR sequences, richer information on the microstructure and function of the brain. This has overall effects on the type and quality of data that can be collected. Figure 2 demonstrates the clear differences in technical quality.

A striking disparity exists in the quality of MRI data obtained from high income countries (such as the HCP data) and that obtained from low-income countries (such as a clinical set up MRI scan in Nigeria; see Figure 2). The HCP data has a higher image quality and spatial resolution (3D) which makes it a better fit for microstructural and functional analysis while the clinical set up scan data has a lower image quality and lower spatial resolution (2D) which is good for clinical diagnostic use but not the best fit for obtaining microstructural and functional brain information. Indeed, as we attempted to use major data tools developed in high-income countries (e.g., Europe, UK and the USA) to pseudonymize or deface the subjects’ data for Wogu et al. (2023), we discovered that none of the tools was designed to handle data such as the one that we were able to collect in Nigeria. As a result, we ended up resorting to manual defacing of the MRI data. In addition, advanced tools designed to segment and classify different tissues and estimate morphometric statistics of the brain (Fischl, 2012) failed to accurately segment and estimate morphometric tissue properties. Finally, attempts to transform the clinical-quality data to research quality data using the most advanced AI algorithms (Iglesias et al., 2023) failed, highlighting potential current biases in training data for the most advanced neuroimaging analysis tools. Whether tools developed in high-income countries should be designed to work on low quality data such as that coming from low-and medium-income countries is a question that the community might need to address. In the future, the scientific community might need to evaluate the potential need for major analysis software to be able to handle data from low-, medium-and high-income countries, especially if these countries start generating data shared globally.

Furthermore, there is a dearth of skills necessary for the use of digital technologies for data generation, processing, application and sharing. As one participant echoed; *“as lecturers we do not have the technical skills and expertise in the use of technologies for brain data to impact on our students. I have tried to collect data from diagnostic centres in Ethiopia, but the radiologists did not know what I was talking about.”* This confirms what we saw on the ground. MRI technicians are not yet trained to collect research data but have experience in clinical scans only. They are often trained to generate images that are good for clinical diagnosis but may not be suitable for advanced research analysis. The lack of focus on research also means that data on patients (including images) are not properly stored or archived. A record keeping culture is often missing; images are deleted due to lack of storage systems. It was also observed that a good number of patients’s brain MRI datasets were saved in formats (XML/AVOL and JPG) not compatible with neuroimaging data analytic tools. Only a limited number of brain MRI data were stored in the appropriate format (DICOM) which limited the quantity of datasets collected.

The training of the young generation is indeed impaired. Lack of knowledge of new experimental approaches, methods of brain mapping and computational and theoretical models is extremely limited, effectively putting Africa several years behind for what pertains to medical imaging and neuroimaging data analysis. Furthermore, the lack of expertise in data, especially statistics and data science within neuroscience and related disciplines, also limits the application of the latest, cutting-edge data analysis approaches to brain data in Africa.

In addition to challenges around the measurement devices, challenges exist even after data collection. For example, the lack of network bandwidth to share data from the imaging centre to the research institutes and universities require that data be collected on CDROM and be transported and saved on personal computers. Hard drives to store data are limited and the computational power to manage, process and analyse large datasets is non-existent. Overall, this situation creates siloed dataset of potential utility that remain closed inside institutional repositories, creating an unFAIR situation for the new generation of African students and researchers.

Finally, the behavioural data sets needed in association with imaging data are still unstructured, manually collected, manually stored often with pen and paper, without digitization. One participant linked the technical challenges to economic challenges; thus, “*you need to spend a lot of money. These devices are not widely available in Sudan, this not only means problems for ageing patients’ due shortage of necessary devices such as MRI, but also for research.”* This demonstrates that some of the technical challenges identified above are linked to micro and macro financial constraints.

#### 4.1.2 Economic challenges

Many of the participants pointed out that economic challenges are critical. According to one participant, *“Africans are too broke to spend money on brain data generation and there is a lack of established funding bodies unlike in Europe and America.”* Data collection requires financial resources that are not available in Africa. As another participant opined; *“I think Africa is full of brilliant minds and the economic barrier is the top reason that is holding us back.”* In other parts of the world evidenced in the formation of the international brain initiatives (IBI) comprising large-scale projects (Adams et al., 2020), there are public and private research grants available for data generation and for infrastructure that can facilitate curation and sharing. These are lacking in Africa. Researchers most often spend their own money and time to acquire brain data. This was the case for us. Our data collection, processing and sharing process were not funded. We funded this with our personal money, and it is an approach that is not sustainable considering the economic situation in Africa. As a participant observed, FAIR brain data; *“would require lots of investment to be sustainable considering the digital infrastructure challenges we face …”* Further connections between economic challenges and technical challenges were also made. One participant observed that; *“the lack of financial means is a real obstacle which prevents us from having equipped laboratories as well as imaging and neurophysiology devices adapted to our needs”* Laboratories in higher institutions in Africa are not well equipped to generate, process, store and share digital data due to lack of funds.

Additionally in clinical settings, inadequate financial resources and an under-resourced health care system contribute to delays in accessing medical facilities and in some cases, absolute inaccessibility to these healthcare facilities and services. As one participant said, *‘even the diagnostic centres do not have sufficient data because not many patients can pay for the high cost of scanning’.* In most African countries, the available health insurance (subscribed to by a handful of people) does not cover the cost of neuroimaging services. Patients often pay out of their pockets and the costs are high. That contributes to a dearth of scans available for research from hospitals and diagnostic centres.

#### 4.1.3 Socio-cultural challenges

As a participant observed, the socio-cultural contexts and interests in Africa prevents the prioritisation of FAIR brain data. This was captured in this response; *“The behaviour and cultural thinking affects the actions, abilities and interpretation of how people see the world. Thus, the African socio-culturally is engaged with other problems and this compounds their basic problems towards action and behaviour. Finally, making them understand FAIR as a problem is difficult to do.”*

It is also identified that a number of neurological disorders are not considered clinical problems that require clinical solutions or research owing to socio-cultural beliefs, perceptions and values. One participant observed; “*oftentimes, Parkinson Disease (PD), Alzheimer’s and other mental disorders associated with cognitive decline which often occur in elderly people are considered as part of the ageing process which do not require treatment.”* The extreme version of this belief is that the person is considered as mentally deranged. “*We still regard people as mad people that belong to the marketplace”* as a participant observed. The implication of these is that families generally feel that there is nothing they can do in terms of treatment and clinical care. It is important to note that in African culture, elderly people, especially those with declining mental capacity are communally cared for by family members. It is believed that love, care and companionship are what the sick aged persons need at this phase of their lives. These perceptions mean that less people seek clinical answers to possible cases of neurodegenerative diseases. The effect of this is that only a small number of MRI scans are conducted in hospitals and private diagnostic centres.

These beliefs can be religious beliefs which affect African understanding of brain diseases and corroborates what Okpalauwaekwe et al. (2017) observed in literature that, it is common to attribute mental illness to supernatural factors such as witchcraft, ‘juju’ (sorcery), evil spirits, divine punishment. These beliefs drive patients with mental health diseases such as major depressive disorders, schizophrenia, psychosis and epilepsy and their families to religious houses, rather than seek medical solutions. Prayer houses and healing or herbalist centres are major places many people seek remedies. It is often difficult to convince them otherwise because they have been indoctrinated in their beliefs and perceptions. This belief in supernatural causes of mental health disorders exists among people of different educational status. In a case study presented by Asare and Danquah (2017) caregivers of rich and well-educated families attributed dissociative amnesia to spiritual causes. Moreso, deep cultural and religious beliefs in the reincarnation also affect tissue donations. This makes the establishment of open access biobank difficult. Reincarnation is the belief that at death, the soul is reborn in new physical forms and any form of mutilation of the body can affect the rebirth of the soul. This belief influences people’s rejection of postmortem examination as well as organ donation (Ulasi et al., 2020). Many cultures in Nigeria and African in general have cultural or religious beliefs in reincarnation, which prevents human tissue donations. That means that there are no available tissue banks that can inform diagnosis and research into many neurodegenerative diseases in Africa.

Huge amounts of brain research in Europe and the United States rely strongly on deceased brain and spinal cord tissues donated by the public for diagnosis and research. There are a good number of brain or bio banks that form good resources for brain research that demonstrate this point. For instance, the Brains for Dementia Research initiative has been founded and funded jointly by Alzheimer’s Society and Alzheimer’s Research UK to support brain donation and provide much-needed brain tissue for researchers (Francis et al., 2019). And since its establishment, the initiative has supplied over 80,000 samples of brain tissue for research and these have led to impressive advances including advances to improve the diagnosis of vascular dementia and the identification of new risk factors for dementia (Aoife, 2018). This type of resource that supports research as well as clinical diagnosis is still lacking in Africa.

Another defining socio-cultural factor that affects conduct of brain scans and subsequently the availability of African brain data globally is mistrust. As a participant observed; *“In many African communities, there is a deep-seated mistrust of external entities, especially when it comes to sharing sensitive data such as brain data. This mistrust can stem from historical exploitation and a fear that data might be misused, misinterpreted, or used for purposes that do not benefit the community from which it originated.”* This level of mistrust is at the root of the emerging ecosystem of data localisation policies in Africa (Babalola, 2024).

#### 4.1.4 Ethical and legal challenges

There are also several ethical challenges that influence brain data generation in Africa. According to one of the participants, the ethics approval process is a tedious process; *“in my country, obtaining ethical approval for clinical trials and utilising human data is quite cumbersome with concerns relating to the legal implications.”* Another participant echoed the same sentiment; *“Federal hospitals make it hard because before you can get approval it would take a while. Other research documents would get approval quickly but when it comes to neuroimaging it is always a hassle. You will be asked different unnecessary questions.”* These insights align with data practices on the continent following a number of unethical clinical trials in Africa (Lurie and Wolfe, 1997; Stephens, 2006). There is a strong awareness of the importance of ethical conduct of biomedical research. However, while most institutional research ethics boards do exist, implementation remains questionable. In the clinical setting where we collected data, ethics approval processes were complex, expensive and sometimes unclear. Many of the diagnostic centres requested money to grant ethics approval, even though we had ethics approval from the University Research Ethics Board. This delayed the process unnecessarily and prevented collection from certain identified collection sites.

Additionally, legal issues regarding the processing of brain data in Africa remain foggy without clear regulatory frameworks. One participant noted; *“I know there is a regulation on data but what it means for brain data is what I do not know.”* This means that in many cases, whereas informed consent is obtained as part of the research protocol, consent for further processing and sharing of the data is lacking. This is particularly important in the face of an emerging data protection regulatory ecosystem in Africa.

In many countries of Africa, data protection regulations and laws are beginning to implement data localization provisions (Babalola, 2024). These countries have enacted laws requiring the storage of data locally and cross-border transfers of personal data are banned unless authorised by a data protection authority, designated entities or by the consent of the data subject (CIPESA, 2022). Justifications for this include the need to protect national security (Kugler, 2022), promote the local digital economy, and to ensure adequate data security and users’ privacy (Kimumwe, 2021). Despite the fact that many of these regulations often do not consider the dynamics of data processing in health research (Eke et al., 2021), this affects all brain data considered under the regulations as personal data.

### 4.2 Importance of making African brain data FAIR

In addition to asking questions on challenges, the participants were asked of their views on the need for and importance of making African brain data FAIR. The FAIR Data Principles are a set of guiding principles designed to enhance data findability, accessibility, interoperability and reusability (Wilkinson et al., 2016). In the age of data intensive science, these principles provide guidance for data producers and publishers on how to overcome challenges to making data available for reuse in research and innovation. As mentioned above, the meaning of data in this context goes beyond the conventional idea of data only as raw measurements of nervous system structure, operational properties, and function but includes derived data as well as metadata that describe the full set of processing steps and analyses used to produce derived data (Eke et al., 2021).

These are related but independent principles that provide normative and practical guidance and that define characteristics that data resources, tools, vocabularies and infrastructures should possess to enable secondary reuse. They are principles that target a number of barriers to knowledge discovery and reuse for both humans and for emerging machines used for scientific analysis. These barriers can include but not limited to: technical challenges (e.g., lack of technology for data storage, curation, sharing or analysis, cybersecurity, compatibility issues), socio-cultural challenges (e.g., no incentives to share, organisational or institutional cultures), economic (e.g., lack of funding, funders’ restrictions), legal or political (e.g., data protection laws, political sanctions). Greater discovery of data and wider reusability therefore relies on overcoming these challenges.

According to the participants, there are a number of critical educational, research and innovation implications of making African data FAIR including but not limited to increasing reproducibility, facilitating generalisability of brain research and innovation outcomes, reducing the cost of data collection, ensuring that Africa gains the benefits of other digital technologies such as artificial intelligence etc.

#### 4.2.1 Increasing reproducibility of brain research

According to one of the participants, *“African researchers cannot continue to work in silos. There is a need for reproducibility.”* Neuroscience research is not immune to the reproducibility crisis (Kelly and Hoptman, 2022; Miyakawa, 2020; Poldrack et al., 2017). Reproducibility is a concept that describes the ability of producing the same results from the same data (Nichols et al., 2017). This is different from replicability that refers to the ability of producing consistent results from new data (Klapwijk et al., 2021). Lack of availability of datasets behind published research results is one of the major causes of scientific irreproducibility. That is why FAIR data principles have become a crucial part of general project planning as well as a core funding requirement. Making FAIRness of African brain data will, thus, contribute to the overall scientific reproducibility of neuroscience research conducted in Africa and on Africans.

#### 4.2.2 Generalisability of research outcomes to African population

Scientific Research and innovation are fundamentally shaped by data. As one of the participants acknowledged, FAIR African brain data will help in aligning research and innovation in neuroscience to the needs of Africans; *“without our data, global research outputs may not work for us. The COVID-19 taught us a lot of lessons.”* The degree to which research results or innovations are generalisable to all contexts and populations depends critically on the degree of diversity or heterogeneity of data. Applications of brain data in research and innovation not only lead to understandings of brain structure and function but they also lead to the development of clinical solutions, therapies and medical technologies (Kellmeyer, 2021). Non-representativeness of data from a group or population can lead to biassed or discriminatory research or innovation outcomes. This issue can be seen in racial biases in modern technologies, including eye tracking (Blignaut and Wium, 2014), face detection algorithms (Buolamwini and Gebru, 2018; Furl et al., 2002), and medical artificial intelligence (AI) algorithms (Obermeyer et al., 2019). Neurotechnologies or therapies developed may not be able to work for populations whose datasets are missing research and innovation processes. FAIR African brain data will ensure that African populations are represented in the global brain data ecosystem; making sure that global brain research and innovation outcomes are generalisable to populations in Africa.

#### 4.2.3 Reducing the cost of research

Research is often an expensive endeavour. One of the participants pointed this out; *“data collection is costly. If we can share data, we can save a lot of money.”* A considerable portion of the cost can be attributed to the data creation and collection phase which for brain research often includes the use of expensive equipment and tools such as MRI Scanners and EEG machines. It also includes the time costs for reading, analysing and refining and classifying data to make it understandable and useful for research purposes. Efficient neuro-data storage also results in additional financial costs as in many cases it includes the cost of indexing and querying, and the technical difficulty of big data visualisation (Hider et al., 2022). The application of FAIR principles to research data can enable such costs to be minimised. This is because of the reduced need for the collection of new research data as FAIR encourages data reuse. Also, as FAIR data is well documented, the time lost due to unfindable, unstructured and incomplete data is limited. Similarly, time spent on data analysis by researchers is reduced with the use of relevant software to read, understand and automatically recognise patterns. In fact, a cost–benefit analysis conducted for the European Commission (PwC EU Services, 2018) has shown that the annual cost of not having FAIR research data could cost the European economy at least €10bn each year. This figure gives some indication of the cost savings that could result from making African brain research FAIR.

#### 4.2.4 More efficient and effective diagnosis

*“Our understanding of brain disorders in our communities will surely change if we can share and apply brain data in Africa. Maybe we can help to diagnose better.”* This was observed by one of the participants. Brain research is a data-driven endeavour but despite the advances made over the years, the diagnosis and prognosis in brain related diseases remain unclear and uncertain in many situations. These are areas where the application of FAIR principles can enable improvement; it results in a better quality of data management and amplifies the quality of metadata and datasets along with their machine readability. Like other biomedical fields (Wise et al., 2019) the use of FAIR data makes the data more suitable for use with advanced analytical tools developed with the aid of AI and machine learning. Using such advanced analytical tools can potentially improve the diagnosis and enable a personalised precision approach. Application areas include medical image quantification and analysis, signal processing, automated analysis of genetic data and disease prediction (EPRS, 2022). AI can also be used to better understand diagnosis for example in situations where there are overlapping clinical presentations but different treatment options (Dwyer et al., 2018). However, the potential for AI to support brain research can only be fully realised through the application of FAIR data principles to brain data as this makes them available in suitable formats for use in modern computing environments.

#### 4.2.5 Increasing citations

As one of the participants mentioned, FAIR data can also increase citations. This participant said, *“I also heard that it could help increase citations which is a good thing’.* This aligns with evidence from literature because data citation helps to give proper credit to the creators of the datasets, encourages data sharing, and increases the visibility of the research (Cousijn et al., 2018). FAIR data principles can increase a researcher’s citations, where datasets are treated as citable entities. Researchers can assign persistent identifiers, such as Digital Object Identifiers (DOIs), to their datasets, making them citable in the same way as scholarly articles (Wittenburg, 2019). Also, other principles supported by FAIR such as the use of standardised metadata and open access practices enable researchers to make their data more visible to the scientific community.

By adopting FAIR data principles, researchers make their data more accessible and reusable for the broader scientific community. This increased availability and usability of data can lead to more researchers citing the original work, acknowledging the data contributors, and facilitating a culture of data sharing and collaboration. Nevertheless, while FAIR data can create favourable conditions for increased citations, other factors also come into play, such as the significance of the research, the quality of the data, and the impact of the findings.

#### 4.2.6 Leveraging the benefits of other advanced technologies

FAIR data helps researchers to harness the potential of various technologies, including AI, ML, data analytics, the Internet of Things (IoT), and cloud computing. By using FAIR data researchers can access, integrate, and analyse data effectively, leading to enhanced research outcomes and enabling them to take advantage of the benefits offered by these technologies. However, a participant observed that; *“we have a serious dearth of datasets in Africa. This has a negative effect on the development of AI and ML research in Africa.”* For instance, scientists can enhance their ability to harness the power of AI technologies and utilise them to derive significant insights from their data. One reason for this is the interoperability enabled by FAIR data which makes it easier to combine and integrate different datasets from various sources. When data is interoperable, researchers can leverage AI techniques to efficiently extract insights, identify patterns, and derive new knowledge by analysing diverse datasets together.

Similarly, for IoT technologies where vast amounts of data are generated from interconnected devices and sensors, FAIR data principles also promote data interoperability, enabling researchers to integrate and analyse IoT data alongside other data sources. By making IoT data findable, accessible, and reusable, researchers can gain insights, monitor systems, and develop innovative applications for brain research.

Likewise, FAIR data aligns well with cloud computing, facilitating the storage, sharing, and processing of data within the cloud environment. Cloud platforms provide scalable infrastructure and robust computing power, enabling researchers to efficiently manage substantial datasets. By integrating FAIR data into cloud workflows, researchers gain the capability to leverage the scalability, accessibility, and computational resources of the cloud for advanced data analysis and processing tasks.

## 5 Critical discussions and opportunities to grow the African brain data ecosystem

Having identified critical challenges and opportunities of FAIR African brain data, it is crucial to consider how the challenges can be mitigated, and opportunities maximised. Most of the challenges border on the disparity in data generation in Africa compared to the global north either because of lack of expertise to collect datasets such as MRI for research, lack of funding or infrastructure. We introduce a number of points for diverse stakeholders in academia, policy, industry and patient groups that need to be considered in building efforts to make African brain data FAIR. These include building a sustainable and inclusive human and technical infrastructure, creating functional funding schemes and developing effective policies and regulations.

### 5.1 Capacity building

People with required skills and expertise are central to FAIR and data processing in general. Data is only made FAIR and kept FAIR by people with the right skills. To address the global burden of neurological, neuropsychiatric, substance-use and neurodevelopmental disorders, there is a need for a functional research ecosystem with trained/skilled scientists and clinicians who can generate, process, apply and share the right brain data for research, practice and policy. Lack of such capacity can be blamed for the reduced worsening effects of mental health disorder in low-and-middle-income countries. There is an urgent need to build new, and strengthen existing individual, institutional and national brain data capabilities. Leveraging brain data for accelerated scientific discoveries and innovation in Africa requires capacity building that can ensure integration of appropriate methods, tools and technologies to generate and analyse big brain data.

Each stage of the data lifecycle, from data generation, processing, curation, archiving to sharing demands specific skills and expertise. From experimental design skills, data science and analysis skills, to data standardisation, curation and management skills, maximisation of brain data requires diverse skills. These skills are currently lacking in the brain research ecosystem in Africa. In addition to this data science, which is increasingly becoming a necessary skill in neuroscience, due to the expanding nature of brain data, has not been integrated into the neuroscience or psychology curricula. A new generation of neuroscientists are being prepared without adequate skills to optimally generate or utilise brain data.

Purposeful, systematic and goal-oriented capacity building is needed to strengthen human resources and infrastructure to enable African researchers and institutions to become independent and responsive to the emerging global brain data ecosystem. To be sustainable, inclusive and effective, this must be created with the individual researchers and institutions in mind. Targeted activities such as training and workshops can be used as pathways for the development of necessary capacities within the FAIR brain data ecosystem. Local and international experts can contribute here by establishing capacity building programmes to create and support a network of highly skilled trainers who will effectively adapt and grow the knowledge and skills acquired. This will thus create sustainable capacities within the African brain data ecosystem.

There is also a need to integrate computer science, which is critical to FAIR brain data, into curricula in fields such as neuroscience, psychiatry and Psychology. Computer approaches and digital technologies are crucial to achieving all the elements of FAIR. Indeed, the current global landscape of brain research and innovation is characterised by the convergence of emerging technologies in the different stages of the data lifecycle. It is important therefore, to establish curricula that will lead to the production of capably skilled students. Heads of institutions need to develop pathways of integrating key aspects of computer science into brain education.

### 5.2 Technical infrastructure

Technical Research Infrastructure (RI) to facilitate sharing and reuse of brain data are notably lacking in Africa. RI, i.e., such as compute resources, centralised data repositories, federated learning infrastructures and virtual research environments have become central to establishing the modern neuroscience scientific enterprise (Amunts et al., 2023). RI facilitates compliant data management, processing as well as computational modelling and analysis software development. Examples of RI developed in the United States, Canada and the European Union include data platforms such as brainlife.io (Hayashi et al., 2024), CBRAIN (Sherif et al., 2014) or EBRAINS (ebrains.eu), as well as data archives such as OpenNeuro (Markiewicz et al., 2021), DANDI (dandiarchive.org), DABI (Duncan et al., 2023), or BossDB (Hider et al., 2022). These archives and platforms are available world-wide under specific user access policies allowing the utilisation of resources that can be and have been used by African researchers and trainees. The development of BIDS metadata standard (Gorgolewski et al., 2016) and the continued work of the International Neuroinformatics Coordinating Facility (INCF) (De Schutter, 2009) in promoting standards for Open, FAIR, and Citable neuroscience are critical to the current and emerging trends in FAIR neuroscience data.

But Africa needs locally developed and supported RIs for several reasons. The first reason is the nature of regulatory frameworks for data protection that exists in Africa. Many African countries have data localisation provisions in their data protection laws or regulations. This means that personal data are not allowed to be transferred outside the jurisdictions they were generated from. Making datasets available for downloads outside of Africa may prove legally difficult or impossible in some cases. Therefore, there is a need to develop virtual research environment (VRE) RIs that can facilitate reuse without compromising the privacy and confidentiality of the research participants. RIs developed for Africa that is sensitive to African ethical and regulatory provisions and principles are needed to make African generated brain data FAIR. The second reason is that an RI that is developed and maintained locally will contribute to furthering African interests and contexts. Like any technology, who owns and controls RIs shape what impacts they can have. Being able to control the narrative on impact especially for Africans is critical to making RIs for FAIR African brain data responsible and sustainable.

However, RIs are not the only technical infrastructure needed for FAIR African brain data. Brain data generation requires a variety of different tools and techniques for different tasks and neural recording. In Africa, there is a dearth of these tools for collecting brain data such as MR Scanners, PET machines, EEGs systems, MEGs scanners, proprietary software for data analysis or collection are also lacking due to high costs.

### 5.3 Funding

Ensuring a sustainable and efficient FAIR brain data ecosystem requires robust funding; for acquiring tools for data generation and storage, development and maintenance of RIs and technologies for interoperability and reuse. However, Africa has a long history of poor funding for research and innovation. For instance, as at 2019, Africa’s funding for research and development was 0.42% of national GDPs which is lower than the global average of 1.7% (Adepoju, 2022). Funding for research is not often prioritised by government agencies. Higher education institutions lack crucial funding and resources to create functional research laboratories, procure research infrastructure and employ capable and experienced researchers. According to Bothwell (2018), half of academics in Africa receive no research funding. Such a low level of funding for research and innovation is demonstrated in the lack of research and innovation outputs from Africa including the lack of production of health technologies (Omotosho et al., 2019) and vaccines (Irwin, 2021). FAIR African brain data can become a catalyst for increased application of data that can lead to effective health technologies and clinical therapies sensitive to African population contexts and needs. Therefore, it is imperative that it should receive sufficient funding for individual researchers as well as institutions.

In the last few decades, the African research and innovation ecosystem has relied heavily on funding from outside the continent. This means that most research priorities for Africa are often decided by overseas funding organisations (Ngongalah et al., 2018). In relation to data, this needs careful consideration in terms of who owns and controls African Brain Data. Funding schemes for FAIR African data needs to be structured in a way that ownership and control of the data remains in the hands of Africans. Infrastructures for storage, archiving and access to African brain data needs to be African centric to reduce the possibility of data colonialism.

It is also important for the unsustainable national and regional research funding structures to be re-examined. African public and private entities need to start the prioritisation of research and innovation. This needs to start with funding for increased STEM education to funding for broader research agenda in African Universities. It is the only way Africa can become competitive in the global innovation ecosystem.

### 5.4 Policy and regulatory reviews and initiation

Africa does not lack research and education policies and regulations. What is lacking is the effective implementation of these policies in ways that can promote advancements in the research ecosystem. For instance, Fosci et al. (2019) reported that Nigeria has a proliferation of competing sectoral policies and strategies for research and innovation. For Nigerian researchers in neuroscience, there are the National Health Act of 2014, the National Health Policy of 2016, Data Protection Act, 2023 and the recently signed Mental Health Act, 2023 to consider. Even though some of these regulations acknowledge research, they often do not provide clear and specific guidelines on research activities including data processing. This lack of clarity, combined with poor mechanisms to ensure their implementation negatively affect the research system by creating overlapping policies, fragmented activities and failure to achieve synergies with national research objectives. The lack of a harmonised approach to research in regulatory documents means that they have limited impact on the national and regional research ecosystem. Most importantly, there is neither an imperative nor incentive for in policy for researchers to make data FAIR. There is a need to establish a regulatory landscape that can effectively shape health research and that emphasises and strengthens FAIR data activities and institutions. This includes institutional policies and practices relevant for inclusive, equitable and transparent research that involves greater data generation, processing and application. In this regard, Africa also needs to consider policies and regulations on animal use in research that can measure up to international standards to prevent ethics dumping (Eke et al., 2023; Eke, 2024). Chatfield et al. (2021) have reported that ethics dumping - the practice of undertaking research that is legally and ethically not permitted in your country in another country that can permit such research - is a major challenge for research ethics committees in Kenya. African countries need to set up protocols that can help to maintain responsible generation of animal data.

## 6 Conclusion

In this paper, we have highlighted the challenges FAIR brain data faces in Africa as well as the opportunities it presents to brain research and innovation ecosystem in the region. The four categories of challenges (socio-cultural, economic, technical, ethical/legal) often form barriers to brain data generation, processing, sharing and application. FAIR starts with data generation. If data is not generated, there will be nothing to share. In Africa, FAIR brain data challenges start with lack of generation due to lack of necessary expertise and resources (including funding and tools) for data generation.

However, when these barriers (pointed out above) are mitigated, reproducibility and generalisability are improved, costs of research are reduced, and there will be more available African brain datasets for innovation. This is critical to the future of neuroscience or brain research and innovation in Africa. We argue that different levels of institutional actions and initiatives are required to grow the FAIR brain data in Africa. Institution here means Africa universities, African academic/professional societies, research centres, government bodies, funders and policy makers. Actions and initiatives on FAIR brain data in Africa should be led and maintained by Africans and for Africa. Whereas non-African or foreign institutions are critically needed for technical support and funding, these need to leverage on fundamentally African platforms, approaches and initiatives. This way, practical approaches for FAIR brain data in Africa will be built on African principles, values and cultural contexts and to provide solutions to unique African neuroscience questions.

For individual researchers who are looking for a pathway to establish FAIR data pipelines or workflows, here are some useful tips from our experience. The first thing is to show feasibility for collecting data. This may involve putting together a team, dedicating sufficient time and infrastructure to collect a small amount of data (any type of data from humans or non-human animals). An example of this may be to find a willing diagnostic centre or hospital where you can set up informed consent protocol for data collection after obtaining ethics approval from relevant institutions. Secondly, you need to find a secure infrastructure to store/curate the data. If the datasets include personally identifying information of living patients, there is a responsibility to protect their privacy and confidentiality. Publish a data sharing paper from the data collected which can become a proof of concept for grant application for larger data collection. The idea for the brain data ecosystem is to aim big but start small. One organisation that is strongly promoting this is the African Brain Data Network (ABDN)—a network of African researchers dedicated to advancing sustainable brain research, education, and innovation in Africa through the responsible collection, processing, sharing and use of big brain data. African researchers need to work together as a community to build a thriving FAIR brain data ecosystem that is socially acceptable, ethically responsible, technically robust and legally compliant. Collaboration is the key. This will not only profoundly benefit Africa but the global brain research and innovation ecosystems.

Nevertheless, it is important to note that whilst 59 responses from the survey provided sufficient statistical rigour for this paper, it does not represent the full landscape of neuroscience research and practice in Africa. According to Aderinto et al. (2023), neuroscience research in Africa has shown a persistent upward trend in recent years, particularly with the emergence of Society of Neuroscientists of Africa (SONA) and the financial support from International Brain Research Organisation (IBRO). Two decades of neuroscience publication trends in Africa as reviewed by Maina et al. (2021) also confirm this upward trend. Information on SONA^2^ and IBRO^3^ websites show that there are currently about 23 countries with affiliated national associations (11) or individual members of both associations (SONA and IBRO). These show that in future research, more responses from the neuroscience community will be needed to provide more statistically reliable data.

## Data Availability

The primary data that informed this paper have been made open and can be found here: http://doi.org/10.17639/nott.7508. Further inquiries can be directed to the corresponding author.

## Footnotes

^1^ https://zivahub.uct.ac.za/

^2^ https://sonafrica.org/affiliate-societies/

^3^ https://ibro.org/about/governance/membership/
